# m6A methylation: Critical roles in aging and neurological diseases

**DOI:** 10.3389/fnmol.2023.1102147

**Published:** 2023-02-21

**Authors:** Yishu Fan, Xinyi Lv, Zhuohui Chen, Yanyi Peng, Mengqi Zhang

**Affiliations:** ^1^Department of Neurology, Xiangya Hospital, Central South University, Changsha, China; ^2^National Clinical Research Center for Geriatric Disorders, Xiangya Hospital, Central South University, Changsha, Hunan, China; ^3^Hunan Key Laboratory of Medical Epigenomics, Department of Dermatology, The Second Xiangya Hospital of Central South University, Changsha, China

**Keywords:** neurovascular unit, 6-methyladenosine, CNS diseases, RNA methylation, m6A

## Abstract

N6-methyladenosine (m6A) is the most abundant internal RNA modification in eukaryotic cells, which participates in the functional regulation of various biological processes. It regulates the expression of targeted genes by affecting RNA translocation, alternative splicing, maturation, stability, and degradation. As recent evidence shows, of all organs, brain has the highest abundance of m6A methylation of RNAs, which indicates its regulating role in central nervous system (CNS) development and the remodeling of the cerebrovascular system. Recent studies have shown that altered m6A levels are crucial in the aging process and the onset and progression of age-related diseases. Considering that the incidence of cerebrovascular and degenerative neurologic diseases increase with aging, the importance of m6A in neurological manifestations cannot be ignored. In this manuscript, we focus on the role of m6A methylation in aging and neurological manifestations, hoping to provide a new direction for the molecular mechanism and novel therapeutic targets.

## 1. Introduction

Epigenetic modifications are crucial posttranscriptional regulations of gene expression, which play important regulatory roles in organogenesis, homeostasis and pathological process (Wang Y. et al., 2021). Since 2013, epigenetic alterations have been listed as one of the nine hallmarks of aging and important changes of cellular senescence (López-Otín et al., 2013; Hernandez-Segura et al., 2018). RNA modifications are important parts of epigenetic modifications. At present, over 160 types of chemical modifications have been identified in RNA, which participate in the regulation of the structural properties of RNA or changing the affinity of mRNA for ribosomes (Zhao et al., 2020). Among all the modifications, N-Methyl adenosine (mA) is the most prevalent internal one and has been found to be highly conserved and hard-coded in mammals and other eukaryotic species (Huang et al., 2020). Studies have shown that N6-methyladenosine (m6A) is abundant in the brain and is involved in the regulation of brain volume, memory formation and consolidation and mammalian postnatal cortical neurogenesis (Ma et al., 2018). Abnormal RNA m6A methylation level is associated with aging associated central nervous system (CNS) changes and the onset and prognosis of various neurological diseases, such as transient focal ischemia, ischemic stroke, Parkinson’s disease (PD), Alzheimer’s disease (AD), multiple sclerosis, depression, epilepsy, and gliomas, etc. (Chokkalla et al., 2019; Chang et al., 2022; Li et al., 2022; Zhang N. et al., 2022).

Aging is a natural process of organismal decay, which is characterized by the functional decline of tissues and organs and the increased risk of aging-associated disorders (Zhang et al., 2020). Brain aging is a complex process, which influences brain structure and functional connectivity (Damoiseaux, 2017). Morphologically, brain aging is characterized by volume loss, cortical thinning, white matter degradation, loss of gyrification, and ventricular enlargement. Pathologically, brain aging is associated with neuron cell shrinking, dendritic degeneration, demyelination, small vessel disease, metabolic slowing, microglial activation, and the formation of white matter lesions (Blinkouskaya et al., 2021). Mechanisms under these changes are not clear, resulting in the lack of effective therapeutic methods (Hou et al., 2019). Epigenetic alterations have been regarded as important hallmarks of aging and cellular senescence (López-Otín et al., 2013; Hernandez-Segura et al., 2018). Considering the abundance and aging related changes of RNA m6A methylation in CNS, it must play critical roles in aging and degenerative neurological diseases. Altered m6A methylation modifications and mutated RNA m6A methyltransferases are associated with diverse neurological pathological processes, which provide new aspects for brain aging research.

**Figure 1:**
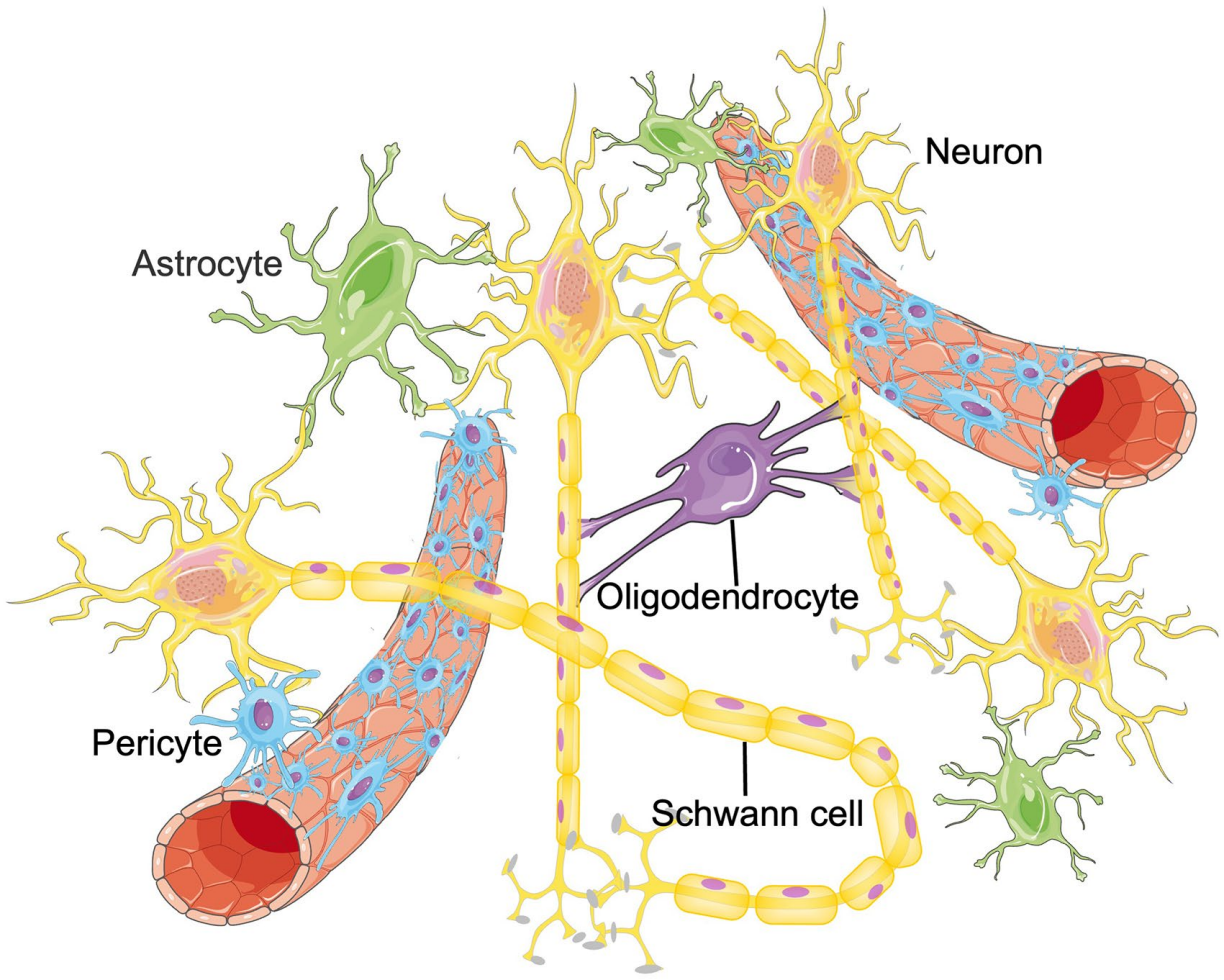
The structure of NVU. The neurovascular unit (NVU) is the minimal functional unit of the brain, consisting of astrocytes, pericytes, microglia, neurons, oligodendrocytes and endothelial cells. The crosstalk between these cellular networks and surrounding extracellular matrix (ECM) plays an essential role in the onset and progression of central nervous system diseases.

The CNS is a complex regulatory network that requires the homeostasis and functional connectivity between neurons and other constituents such as endothelial cells, astrocytes, pericytes, microglia, oligodendrocytes, basement membrane, as well as surrounding extracellular matrix (ECM; Edison, 2020; Schaeffer and Iadecola, 2021). To describe the situation in the brain more realistically, the concept of neurovascular unit (NVU) was proposed, which is composed of neurons, blood–brain barrier (BBB), microglia, pericyte, astrocyte and surrounding ECM, etc. (Seo et al., 2021; Figure 1). Intercellular communication and signaling within the NVU is fundamental to the CNS homeostasis and function (Zagrean et al., 2018). Functionally, the NVU is responsible for maintaining the integrity of the BBB, regulating the cerebral blood flow (CBF) and promoting the signal transmission of local neurons (Yu et al., 2020). Some internal and external factors, such as ischemic stroke or age-related degeneration, can disrupt the balance of NVU. If that happens, neuronal cell death, glial reaction, and immune cell infiltration would subsequently occur, resulting in various neurological diseases (Cai et al., 2017a; Wang L. et al., 2021). Therefore, functional recovery of NVU through remodeling these cellular networks have become an emerging therapeutic target for aging related neurological diseases and ischemic CNS diseases (Ozaki et al., 2019; Forró et al., 2021). Published papers have found that RNA m6A methylation participates in promoting angiogenesis and nervous system development, which are important parts of NVU remodeling. Therefore, m6A methylation could be the cross hub linking aging, NVU remodeling and neurological diseases. In this manuscript, we summarize recent findings in the field of RNA m6A methylation and NVU remodeling and discuss the potential application of m6A methylation in the treatment of different neurological diseases. We hope to provide novel therapeutic targets for future disease management.

## 2. The CNS system: Remodeling during aging and diseases

The development of the human CNS requires the precise orchestration and coordination of myriad molecular and cellular processes across a staggering array of cell types and over a long period of time (Bohlen et al., 2019). The formation of the CNS begins early in development with the induction of the neural ectoderm on the dorsal surface of the embryo. Subsequently, the neural ectoderm plate changes its shape to form a neural groove and eventually, a neural tube. The wall of the neural tube is composed of germinal cells, collectively called the neuroepithelium, that produces neurons and glia throughout the CNS (Bayer, 1989). After that, neuron begin to migration, glial cells differentiate and mature, laminar organized and form regional patterning and lateralization of the human neocortex, which facilitates neural circuit assembly, maturation, and plasticity (Silbereis et al., 2016). To better understand the function and structure changes of CNS, the concept of NVU was proposed as a structural and functional unit of brain which is composed of neurons, BBB, microglia, pericyte, astrocyte and surrounding ECM, etc. NVU emphasizes the symbiotic relationship between the nervous system and the microenvironment, which is a dynamic interaction between multiple cells instead of a simple nutritional relationship. The dysfunction of NVU is an important pathological feature underlying neurological disorders and during aging process (Sato et al., 2022). When that happens, cells and other constituents in NVU interact with each other to maintain homeostasis and promote NVU recovery. These changes are called NVU remodeling, which has significant influence on the onset and prognosis of various neurological manifestations (Zagrean et al., 2018).

The process of NVU remodeling involves the interactions between neurons, glial and microvascular cells that create a microenvironment in which neurological recovery may ensue (Zagrean et al., 2018). Take NVU remodeling during ischemia/reperfusion as an example. When ischemia occurs, decreased cerebral perfusion leads to neuronal injury and death, which is the beginning of NVU remodeling. Diverse proinflammatory mediators released by damaged neurons leads to astrocyte end-foot swelling and increased endothelial vesicles (Haley and Lawrence, 2017). Impaired astrocytes secrete proinflammatory mediators that cause further NVU disruption and neuronal injury. Matrix metalloproteinases (MMPs) produced by endothelial cells and pericytes degrade the basement membrane and cause the breakdown of tight junctions (Underly et al., 2017). Microglia translocate to the penumbra and exacerbate BBB breakdown (Yenari et al., 2010). Leukocytes then transmigrate across the breached BBB and cause subsequent neuroinflammation (Zhang et al., 2019). After reperfusion, neuronal networks and brain capillaries start to develop, which involves the migration of neural progenitor cells (NPCs), the remodeling of functional axons and synapses and the formation of ECM (Andres et al., 2011). Angiogenesis is the key and the first step for NVU remodeling after cerebral ischemia, which includes proliferation of vessel composing cells, recruitment of pericytes, coverage of endothelial tube by pericytes, and maturation of neo-vessels (Hatakeyama et al., 2020). Pericytes function as vital modulators in angiogenesis and help remodel the BBB and support the neurogenesis (Cai et al., 2017b). Endogenous vascular endothelial growth factor (VEGF) produced by astrocytes promotes angiogenesis and the proliferation of astrocytes themselves (Krum et al., 2008). NPCs are attracted to the damaged area accompanied with the process of angiogenesis, which initiates the neurogenesis process (Hermann and Zechariah, 2009). Interaction between matrix and receptors on NVU cells regulates cell survival and focal bioavailability of growth factors, which is essential for NVU remodeling processes (Stamatovic et al., 2019). Restoring the function of neurons is the ultimate therapeutic target of ischemic diseases. Neuron function restoration requires the local formation of functional axons and synapses along pyramidal tract (Andres et al., 2011; Reitmeir et al., 2011) and within motor cortex (Clarkson et al., 2010; Hermann and ElAli, 2012). Meanwhile, neurons adjacent to the infarct induce axonal growth (Li et al., 2010; Overman et al., 2012; Li et al., 2015; Joy et al., 2019) and synapse formation (Luke et al., 2004) *via* regulating growth differentiation factor 10 (GDF10), ephrin-A5, and C-C chemokine receptor type 5 (CCR5) signals. Also, new axonal projections are formed, which can project into premotor, motor, sensory (Overman et al., 2012; Li et al., 2015; Joy et al., 2019) and retrosplenial cortices (Brown et al., 2009). The remodeling of ECM mainly depends on the glial cells. *In vivo* and *in vitro* studies have shown that, glial cell regeneration occurs before neuron regeneration. After 24 h of reperfusion, microglia fully enwrap small blood vessels in the peri-infarct region. The remodeling process is regulated by many cytokines, such as VEGF, angiogenin, Netrin-4, etc. (Table 1) They participate in mediating cerebral angiogenesis and restoring the function of neurological diseases (Lemons and Condic, 2006). Administration of exogenous endostatin, an angiogenesis antagonist, can not only inhibit angiogenesis, but also inhibit the migration and survival of newborn neurons (Guo and Lo, 2009; Xiong et al., 2010). In the process of neurons remodeling, VEGF secreted by the proliferating microvascular cells promotes the migration of neural precursor cells and the remodeling of neurons and glial cells (Hermann and Zechariah, 2009).

**Table 1:** Remodeling of NVU.

Cells	Mediators	Results	References
M1-type microglia	TNF-α, IL-1β, IFN-γ, IL-6, iNOS, MMP9, MMP3	Promoted inflammatory reactions	Yenari et al. (2010)
M2-type microglia	TGF-β, IL-10, IGF, VEGF	Promoted angiogenesis and suppressed inflammatory reactions	Ponomarev et al. (2013)
M2a-like microglia	IL-4, IL-13	Stimulated tissue repair, immunity against parasites, and growth	Colton (2009)
M2c-like microglia	TGF-β	Tissue remodeling after inflammation subsides	Colton (2009) and Chhor et al. (2013)
Microglia	IL-1β, TNF-α, IL-6, MMP	Disrupted BBB integrity	Pan and Kastin (2007)
	TGF-α, IGF-1	Enhanced neural proliferation and differentiation	Choi et al. (2017) and Thored et al. (2009)
	VEGF	Reconstruction of cerebral blood vessel	Zhang et al. (2000) and Xie et al. (2013)
	CX3CR1	Promoted synaptic pruning	Lauro et al. (2015) and Wu et al. (2015)
	IL-1β	Promoted astrocytic activation, which leads to astrogliosis	John et al. (2004)
	TNF-α	Killed oligodendrocytes	Zajicek et al. (1992)
	CX3CR1	Engulfed endothelial cells	Jolivel et al. (2015) and Lou et al. (2016)
Endothelial cells	BDNF	Promoted neuronal survival	Ward et al. (2019)
Pericytes	PDGF-β	Induced cell growth and anti-apoptotic responses	Arimura et al. (2012)
	MMP9	Damage of tight junction complexes and plasma leakage at places where pericyte somata adjoined the capillary wall	Underly et al. (2017)
Astrocytes	VEGF	Induced angiogenesis, increased astrocyte proliferation and facilitated expression of growth factors	Krum et al. (2008)
	GDNF	Promoted neuronal survival and brain repair	Zhang et al. (2021)
	S100B	Counteracted the stimulatory effect of neurotoxins on microglia and facilitated glutamate uptake	Reali et al. (2005) and Tramontina et al. (2006)

TNF-α, tumor necrosis factor α; IL, interleukin; IFN, interferon; iNOS, inducible nitric oxide synthase; MMP, matrix metalloproteinase; TGF-β, transforming growth factor β; VEGF, vascular endothelial growth factor; IGF, insulin-like growth factor; CX3CR1, CX3C chemokine receptor 1; S100B, S100 Ca^2+^-binding protein B; BDNF, brain derived neurotrophic factor; GDNF, Glial cell line-derived neurotrophic factor; PDGF, platelet-derived growth factor.

## 3. Basic science of RNA m6A methylation

The dynamic nature and increasing number of RNA modifications provide new possibilities for adapting to specific environments by changing gene expression rapidly. At present, over 160 types of chemical modifications have been identified in RNA (Zhao et al., 2020), among which RNA methylation is the most abundant modification. The most abundant and diverse epigenetic modification of mRNAs in eukaryotes is m6A methylation which mainly modified mRNA and lncRNA (Huang et al., 2020). Considering that RNA methylation play an important role in nervous system development (Widagdo and Anggono, 2018) and angiogenesis (Qin et al., 2020), it may play an important role in NVU remodeling.

M6A methylation, first reported in 1974, plays a conservative role in the evolution of meiosis and cell differentiation (Desrosiers et al., 1974; Yue et al., 2015). The abundance of m6A methylation varies in different organs, tissues and cell lines but peaks in the brain (Meyer et al., 2012; Chang et al., 2017). Molecularly, m6A mainly locates near the termination codon of the protein coding sequence (CDS) of the mRNA and the 3 ‘untranslated region (3′UTRs; Dominissini et al., 2012; Ke et al., 2015). The specific modification site of m6A methylation is mainly on the adenine of the RRACH sequence (R is guanine or adenine, A is adenine, C is cytosine, H is uracil, adenine or cytosine), which regulates the stability, location, transportation, splicing and translation of RNA at the post-transcription level (Deng et al., 2018). According to recent studies, m6A methylation plays an important role in multiple processes including mRNA splicing regulation (Haussmann et al., 2016; Lence et al., 2016), mRNA translatability and stability (Bodi et al., 2015; Liu et al., 2017), and alternative polyadenylation site selection (Ke et al., 2015), etc.

**Figure 2:**
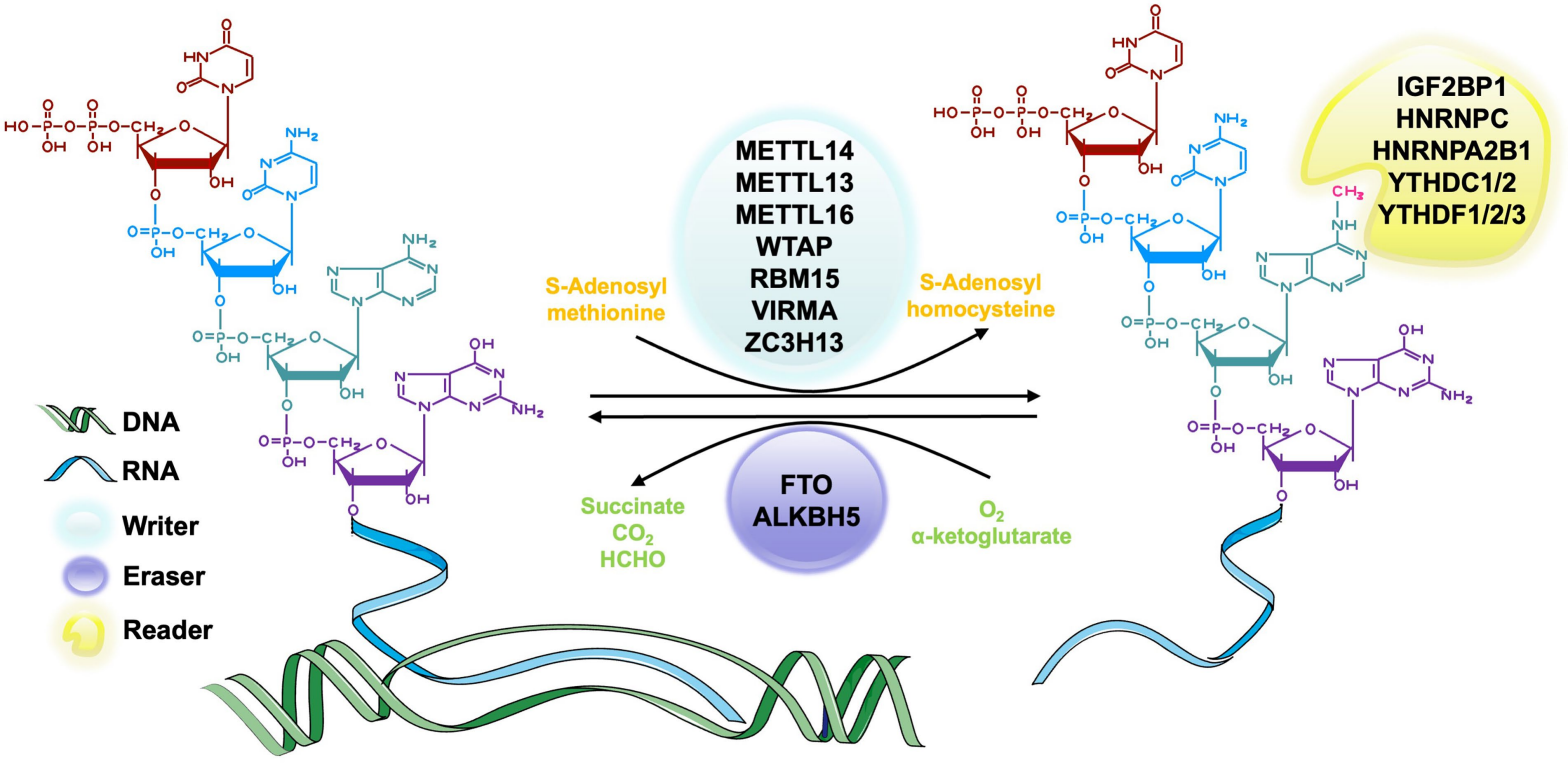
The process of m6A RNA methylation. The m6A methyltransferase, also called writers, catalyze the transfer of methyl groups from the donor S-adenosylmethionine (SAM) to the adenine nucleotides in the recipient RNA subunit. The m6A demethylase, also known as erasers, is responsible for removing the SAM on the adenine nucleotide of m6A-containing substrates. m6A methylation recognition proteins, which are known as readers, function in recognizing and binding to the m6A modified site and decoding the methylation code by recruiting or weakening the connection between the target RNAs and RNA binding-proteins (RBPs) of different functions.

In the process of RNA methylation, three types of molecules are involved: writers, erasers, and readers. Writers refer to methyltransferase, which can add methylation modifications to RNA and mediate the process of RNA methylation modification (Zhou et al., 2021a; Satterwhite and Mansfield, 2022). Erasers are demethylase that erase the RNA methylation modification and mediate the process of RNA demethylation modification (Qu et al., 2022). Readers, known as m6A methylation recognition protein, can read the information of RNA methylation modification, guiding, and participating in the translation and degradation of the downstream sequence of the modified RNA (Wei et al., 2022). These three types of molecules are indispensable for RNA methylation regulation and are powerful tools in the studies of specific mechanism, physiological and pathological role of RNA methylation (Figure 2).

The m6A methyltransferase complex, also known as writer, is responsible for catalyzing the transfer of methyl groups from the donor S-adenosylmethionine (SAM) to the adenine nucleotides in the recipient RNA subunit (Bokar et al., 1997). M6A methyltransferase complex consists of METTL3 (McGraw et al., 2007), METTL14 (Liu et al., 2014), Wilms’ tumor 1-associated protein (WTAP; Ping et al., 2014), vir-like m6A methyltransferase-associated protein (VIRMA, originally known as KIAA1429; Yue et al., 2018), and RNA binding motifs protein 15/15B (RBM15/15B; Patil et al., 2016; Chen X.-Y. et al., 2019). The most common molecular components of this complex are METTL3 and METTL14 (Liu et al., 2014). METTL3 is a highly conserved catalytic subunit and the core component of this complex, which has the ability to bind SAM (Lin et al., 2019). METTL14 is highly homologous to METTL3 and can combine with METTL3 to form a stable heterodimer and greatly enhance the catalytic activity (Liu et al., 2014). METTL3 and METTL4 maintain the main function of this complex together (Liu et al., 2016; Wang et al., 2017a). WTAP is also a core component in the m6A methyltransferase complex that can interact with METTL3-METTL14 complex in catalyzing methylation (Ping et al., 2014). The zebrafish embryos with WTAP knockdown can reduce the abundance of m6A methylation in cells significantly (Ping et al., 2014). It may result from the ability of WTAP to bind the alternative splicing pattern of mRNA (Ping et al., 2014). KIAA1429 is a homologous protein of the Virilizer protein that participates in regulating the catalytic activity of the methyltransferase complex by aggregating the core components (Yue et al., 2018). There are also some other relevant molecules of writers, such as METTL5 (van Tran et al., 2019), tRNA methyltransferase 11-2 (TRMT112; van Tran et al., 2019), Cbl proto-oncogene like 1 (CBLL1; Figueroa et al., 2009) and so on.

The m6A demethylase, also known as erasers, is responsible for removing the SAM on the adenine nucleotide of m6A-containing substrates. According to current research, two m6A demethylases, fat mass and obesity-associated (FTO; Jia et al., 2011) and a-ketoglutarate-dependent dioxygenase alkB homolog 5 (ALKBH5; Zheng et al., 2013), have been identified in eukaryotes. These two molecules belong to the AlkB family and have similar catalytic centers, but differ in their substrates and positioning (Zou et al., 2016). FTO is the first discovered RNA deacetylase and mainly catalyzes RNA demethylation with its C-terminal structure in the nucleus (Bartosovic et al., 2017). Studies showed that FTO played an important role in neural differentiation (Li L. et al., 2017), lipogenesis (Zhang et al., 2015) and bone mesenchymal stem cells (BMSCs) ossification (Wang et al., 2017b). ALKBH5 is the second identified m6A erasers in mammalian. It has the similar catalytic ability to FTO (Zheng et al., 2013) but has different organ distribution. FTO has an abundance in the brain (Aas et al., 2017), but ALKBH5 is mainly found in the tests and has an important effect on spermatogenesis (Zheng et al., 2013).

m6A methylation recognition proteins, which are known as readers, functions in recognizing and binding to the m6A modified site and decoding the methylation code by recruiting or weakening the connection between the target RNAs and RNA binding-proteins (RBPs) of different functions (Roost et al., 2015; Spitale et al., 2015; Adhikari et al., 2016; Maity and Das, 2016; Wu et al., 2017). The most important reader in eukaryotes is a group of proteins that have YT521-B homology (YTH) domains. These domains include conserved C-terminal for RNA recognition and the variable N-terminal for RNA binding, which are also considered to be the most primitive m6A readers (Zhang et al., 2010), YTHDF family, including YTHDF1, YTHDF2, YTHDF3 in the cytoplasm, and YTHDC1, YTHDC2 in nucleus (Wang et al., 2014, 2015; Zhou et al., 2015; Li L.-J. et al., 2018) all belong to this group. YTHDF proteins regulate the metabolism of RNA cooperatively (Li A. et al., 2017; Shi et al., 2017). Other readers include but not limited to heterogeneous nuclear ribonucleoprotein A2B1 (HNRNPA2B1; Alarcón et al., 2015), heterogeneous nuclear ribonucleoproteins C (HNRNPC; Liu et al., 2015), heterogeneous nuclear ribonucleoproteins G (HNRNPG; Liu et al., 2017), fragile X messenger ribonucleoprotein (FMRP; Edupuganti et al., 2017), insulin-like growth factor 2 mRNA-binding protein1-3 (IGF2BP1-3; Huang et al., 2018) and eukaryotic initiation factor 3 (eIF3; Meyer et al., 2015).

## 4. Effect of RNA m6A methylation

The m6A modification can regulate gene transcription, influencing the cellular location, stability and translation efficiency of targeted coding and non-coding RNAs (Zaccara et al., 2019). For example, m6A methylation participates in the pre-mRNA spicing (Berulava and Horsthemke, 2010; Zheng et al., 2013; Liu et al., 2014; Alarcón et al., 2015), mRNA stability (Batista et al., 2014) and the translation process (Li L.-J. et al., 2018) of coding RNA. As for noncoding RNA, such as rRNA, miRNAs and lncRNA, m6A methylation can increase the translation efficiency of rRNA (van Tran et al., 2019), influence the pre-rRNA processing of snoRNA (Sergeeva et al., 2020), re-miRNA and lncRNA (Fazi and Fatica, 2019). RNA methylation shows a significant effect on regulating gene expression efficiency. According to recent studies, m6A methylation and other related molecules such as FTO and NSun2 are abundant in brain (Blanco et al., 2011; Lence et al., 2016; Aas et al., 2017; Chang et al., 2017). These molecules play an important role in the differentiation and growth of nervous system and blood vessels. Following, we introduce the effect of RNA methylation in NVU remodeling from the aspect of blood vessel repair, neurons regeneration and other CNS cell function.

### 4.1. RNA m6A methylation in blood vessels repair and angiogenesis

Cell differentiation, especially mesenchymal stem cells (MSCs) and hematopoietic stem cells (HSCs), is a key process in blood vessels repair and angiogenesis, which is also a new therapeutic target of ischemic stroke (Hao et al., 2014). m6A methylation plays an important role in various developmental decisions including angiogenesis (Mathiyalagan et al., 2019). Studies using zebrafish showed that RNA m6A methylation determines the differentiation stage of cells during the endothelial-to-hematopoietic transition (EHT) process in the development of embryos. This is achieved *via* the continuous activation of Notch signal transduction mediated by the m6A methyltransferase METTL3 in arterial endothelial cells (Zhang et al., 2017; Lv et al., 2018). In malignant hematological diseases, such as acute myeloid leukemia (AML), the quantity of the mRNA modified by METTL3 in blood cells is significantly increased (Vu et al., 2017). Rong et al. found that the density of FTO protein in cells was negatively correlated with micro-vessel density (MVD; Rong et al., 2019). Zhu et al. showed that total Panax notoginseng saponin (TPNS) can modulate the WTAP/p16 signaling axis through m6A modification in vascular smooth muscle cell (VSMC). Increased m6A modification also inhibited vascular intimal hyperplasia, intravascular smooth muscle migration and hyperplasia potential (Zhu et al., 2020).

RNA methylation also regulates angiogenesis and blood vessel repair. *In vitro* studies shows that RNA methylation affects the endothelial cells viability, proliferation, migration, and tube formation (Goyal and Goyal, 2019). Studies showed that m6A methylation level was significantly upregulated in endothelial cells following hypoxic stress and had positive effects on blood vessel repair and angiogenesis (Wang L.-J. et al., 2020; Yao et al., 2020). For example, METTL3 promotes angiogenesis *via* up-regulating putative arterial endothelial marker, hairy and enhancer of split-related with YRPW motif 2 (HEY2), which plays an important role in the formation of capillary-like tubes and endothelial cell migration (Yao et al., 2020).

RNA methylation also promotes angiogenesis *via* affecting cytokine secretion such as angiogenic growth factor (Zaitseva et al., 2019). METTL3 can promote angiogenesis by catalyzing m6A methylation and improving the stability of hepatoma-derived growth factor (HDGF) mRNA (Wang Q. et al., 2020). IGF2BP3 can recognize and bound to the m6A methylation on the mRNA of HDGF and VEGF mRNA. This combination leads to increased expression and stability of HDGF and VEGF and subsequently promote the angiogenesis (Wang Q. et al., 2020; Yang et al., 2020). METTL14/ALKBH5 are also proven to be important molecules that affect angiogenesis. They constitute a positive feedback loop with the RNA stability factor HuR, and promotes epithelial-mesenchymal transition by activating the gene expression of transforming growth factor β (TGF β) signaling pathway (Bertero et al., 2018). Hypoxia is found to reduce the m6A methylation and angiogenesis effects of METTL14/ALKBH5 by affecting the activity of related molecules (Panneerdoss et al., 2018).

### 4.2. RNA m6A methylation in neuron regeneration

According to current studies, the effect of RNA methylation in nervous system regeneration and repair is mainly achieved *via* promoting neuronal development and repair. m6A modification played an important role in regulating gene expression and cell differentiation of NPCs (radial keratinocytes; Yoon et al., 2017) and adult neural stem cells (aNSCs; Li L. et al., 2017) in the process of neurogenesis in the mammalian brain. For example, m6A modification can prolong cell cycle and delay cellular differentiation by promoting the decay of key mRNAs. m6A methylation can also affect the self-renewal, differentiation and lineage determination of various stem cells (Yoon et al., 2017; Wang et al., 2018). m6A methylation is enriched in highly conserved motifs of aNSCs transcriptome. This partially explains its role in promoting the proliferation of aNSCs and the morphological maturation of newborn neurons in the adult brain (Chen J. et al., 2019). By knocking out METTL14 or FTO in mouse embryos, the occurrence of cortical nerves can be delayed, and the development of nervous system is defects to varying degrees (Li L. et al., 2017). A study in *Drosophila* has found that Nito (RBM14 in human) in the m6A methyltransferase complex control and regulate neuronal development activities, such as axon growth and branch, synapse formation by regulating m6A methylation activity in CCAP/bursicon neurons (Gu et al., 2017). Research by Li has shown that conditional exhaustion of the m6A reader protein Ythdf2 in mice can cause irreversible damage in embryonic neuronal differentiation and development, resulting in lethality in the later stages of embryonic development (Li M. et al., 2018).

Neuron injury was found to result in the increased m6A mRNA methylation modification and cellular level of METTL14 and YTHDF1 in adult mice dorsal root ganglia (DRG). Knocking down METTL14 and YTHDF1 genes results in significantly reduced regeneration of sensory axon (Weng et al., 2018). Methylated recognition proteins also play an important role in regulating neurodevelopment. For example, FMRP has the priority to bind RNA probes containing m6A chemical modifications (Edupuganti et al., 2017). FMRP is involved in nervous system development and synaptic plasticity (Hagerman and Polussa, 2015), which is mainly achieved by regulating alternative mRNA splicing, mRNA stability, mRNA dendritic transport and partial post-synaptic local protein synthesis of mRNA (Didiot et al., 2008; Bechara et al., 2009; Ascano et al., 2012; Guo et al., 2015). Some researchers have found abundant m6A methylation on the target mRNA of FMRP (Chang et al., 2017). Moreover, the abundance of FMRP target mRNA in the cytoplasm decreased in FMRP-KO mice, which indicates that FMRP may affect the nuclear export of m6A-modified RNA (Hsu et al., 2019). These studies suggest that methylation recognition protein plays an important role in promoting the differentiation and development of neurons.

### 4.3. RNA m6A methylation in other CNS cells

Glial cells are the most abundant cells and induce several changes in pathological conditions such as inflammation, demyelination and disruption of BBB (You et al., 2022). RNA m6A modification is observed to regulate microglia’s inflammatory processes (Zhang F. et al., 2022). Studies suggest that METTL3 promotes lipopolysaccharide (LPS)-induced microglial inflammation by activating the TNF receptor associated factor 6 (TRAF6)-NF-κB pathway (Wen et al., 2022) and improves neuronal apoptosis and microglial activation by inactivating MyD88/NF-κB pathway (Chen Y. et al., 2022). In addition, m6A reader Igf2bp1 is reported to regulate the inflammatory processes of microglia *via* enhancing the m6A methylation and stabilizing Gbp11 and Cp mRNAs (Ding et al., 2022). It is reported that microRNA-421–3p could prevent inflammatory response in cerebral ischemia/reperfusion injury through targeting m6A reader YTHDF1 to inhibit p65 mRNA translation, which may provide a target for ischemia treatment (Zheng et al., 2020). The m6A methylation also participates in the development of autogenic immune diseases (Zhou et al., 2021b) and the change of dopaminergic neuron function (Teng et al., 2021).

RNA m6A methylation plays an essential role in the development of glia cells and brain tumor (Wang J. et al., 2021). Research conducted by Chang G et al. shows that YTHDF3 promotes cancer cell interactions with brain endothelial cells and astrocytes, BBB extravasation and angiogenesis *via* enhancing the translation of m6A-enriched transcripts for ST6GALNAC5, GJA1 and epidermal growth factor receptor (EGFR), which are all associated with cancer brain metastasis (Chang et al., 2020). Another study suggests that glioma stem-like cells (GSCs) radio resistance is mediated by m6A modification (Visvanathan et al., 2018). Cytoplasmic polyadenylation element binding protein 2 (CPEB2) m6A methylation regulates BBB permeability *via* regulating splicing factor SRSF5 stability, which could serve as a target for improving glioma-specific chemotherapeutic effects (Zhang M. et al., 2022). Besides, m6A regulation is also associated with spinal cord injury (SCI) and may contribute to spinal cord regeneration (Xing et al., 2021). RNA m6A methylation is proven to be crucial for oligodendrocyte maturation and CNS myelination (Xu et al., 2020). Current study shows that Prrc2a plays an important role in oligodendrocyte specification through functioning as a novel m6A reader, suggesting a therapeutic strategy for hypomyelination-related neurological diseases (Wu R. et al., 2019).

Pericytes are also important parts of NVU. m6A RNA modification in pericyte can lead to pericyte dysfunction, which induces vascular complication. Study suggests that METTL3-mediated m6A methylation regulates diabetes-induced pericyte dysfunction, which could be a potential therapeutical target for diabetes-induced retinal vascular complication treatment (Suo et al., 2022). Moreover, the results of m6A high-throughput sequencing suggests that hypertension is potentially involved in the changes in m6A methylation level in microvascular pericytes (Wu Q. et al., 2019). Although there are no studies on nervous system diseases, RNA m6A methylation in pericyte is supposed to play an essential part in NVU remodeling.

## 5. RNA m6A methylation in CNS diseases

NVU homeostasis disturbance and function loss are observed in neurological diseases caused by ischemia (Cai et al., 2017a; Zagrean et al., 2018). The persistent NVU dysfunction is thought to underlie the development of post-traumatic brain injury (TBI) neurodegeneration and late-onset neurodegenerative diseases (Zhou et al., 2020). Therefore, the treatment and recovery also depend on neurovascular remodeling. Research by Chokkalla has shown that compared with the control group, m6A methylation level in the ischemic stroke group increased significantly, mainly through suppressing m6A demethylase (such as FTO; Chokkalla et al., 2019). According to the effect of RNA methylation in the generation and repair of nervous system and blood vessels, RNA methylation can be regarded as a crucial target in the prevention, diagnosis and treatment of related diseases (Wei et al., 2017). For NVU remodeling, nutrition supply is the foremost requirement, which can be achieved by the newborn blood vessels. Considering the promising effect of m6A RNA methylation in angiogenesis, it is possible to be applied to facilitate nervous system repair and regeneration. For example, TPNS can prevent the proliferation of vascular intima and smooth muscle by downregulating m6A methylation level, which can be applied to arterial restenosis (Zhu et al., 2020).

Researchers are making great efforts to seek breakthrough in neuropathy based on RNA methylation and NVU remodeling. Wang et al. revealed the mechanism of m6A modification in regulating angiogenesis and provided potential pharmacological targets to prevent the formation and progression of cerebral arteriovenous malformation (Wang L.-J. et al., 2020). Zhang et al.’s work suggests that the neuroprotective effects of 2-(2-benzofuranyl)-2-imidazoline (2-BFI) in acute ischemic brain damage are at least partly due to the drug’s ability to improve the functions of NVU (Zhang et al., 2018). Brooks et al. found that modulating endothelial barrier function of NVU may provide new therapeutic approaches to improving outcomes in cerebral malaria (Brooks and Hawkes, 2017). However, most studies describe m6A RNA methylation or NVU remodeling separately, but few investigate the combined influence of these two or the mechanisms and effects of m6A in NVU remodeling. More studies are needed in the future to find therapeutic approaches based on the m6A RNA methylation and NVU remodeling.

## 6. Outlook

NVU is a holistic concept that includes cellular components such as neurons, blood vessels, and local microenvironmental components such as ECM and regulatory factors. Previous studies have found newborn neurons in ischemic brain, which can gradually mature over time, replace old neurons in structure and function, and integrate into the neural network to restore brain function (Gu et al., 2000; Zhang et al., 2006). It has been widely accepted that neuroprotective approaches to prevent brain deficits or restore neurofunction should target NVU as a whole rather than neurons alone. Both newborn neurons and neural stem cells count on the special microenvironment created by the surrounding blood vessels and glial cells (Osipova et al., 2018). Existing research confirmed that NVU played an important role in brain development (Yoon et al., 2017), BBB formation and maintenance (Andreone et al., 2015), etc. Targeting NVU remodeling is a promising therapeutic approach for treating cerebral ischemic diseases (del Zoppo, 2010), neurodegenerative diseases (De Strooper and Karran, 2016) and vascular dementia (Iadecola, 2013). RNA m6A methylation is attracting more attention in current gene expression regulation research. At present, many studies focus on the role of RNA m6A methylation in the nervous system or blood vessels, but few on the combination of these two. Meanwhile, studies on RNA m6A methylation in NVU remodeling and brain function restoration is still limited. Finding the relationship and mechanism between NVU remodeling and RNA m6A methylation is of great significance for the prognosis of various nervous system diseases.

RNA m6A methylation related studies in other diseases may provide directions for future research in NVU remodeling and neurofunction recovery. For example, excessive METTL3-mediated m6A modification attenuated the RNA stability of autophagy-related 7 (ATR7) in osteoarthritic chondrocytes. Decreased ATR7 level prevented the formation of autophagosomes and promoted cellular senescence (Chen X. et al., 2022), which could be applied to studying neurodegenerative diseases, such as AD. However, attention also should be paid that reduced expression of methyltransferase complex and subsequent decreased m6A methylation is related to cell proliferation as well as tumorigenicity (Liu et al., 2018). Therefore, a precise spatiotemporal control of m6A methylation is crucial for future clinical application. Orchestrating the m6A RNA methylation level of different cells of NVU is another challenge. Besides, noncell components also influence NVU remodeling. Further studies are needed in this area to provide advanced findings before we can apply to clinical research.

## Author contributions

MZ conceptualized the study, acquired funding, and administered the project. YF and XL wrote the original draft. ZC and YP reviewed and edited the manuscript. All authors contributed to the article and approved the submitted version.

## Funding

This work was supported by grants from the Natural Science Foundations for Excellent Young Scholars of Hunan Province (no. 2021JJ20095), the Key Research and Development Program of Hunan Province (no. 2020SK2063), Research Project on Education and Teaching Innovation of Central South University (no. 2021jy145), the Natural Science Foundations of Hunan Province (no. 2020JJ4134), the National Natural Science Foundation of China (no. 81501025).

## Conflict of interest

The authors declare that the research was conducted in the absence of any commercial or financial relationships that could be construed as a potential conflict of interest.

## Publisher’s note

All claims expressed in this article are solely those of the authors and do not necessarily represent those of their affiliated organizations, or those of the publisher, the editors and the reviewers. Any product that may be evaluated in this article, or claim that may be made by its manufacturer, is not guaranteed or endorsed by the publisher.

## Figures and Tables

**Table:** 

2-BFI	2-(2-benzofuranyl)-2-imidazoline
3′UTRs	3′untranslated region
AD	Alzheimer’s disease
ALKBH5	alkB homolog 5
AML	acute myeloid leukemia
aNSC	adult neural stem cells
ATR7	autophagy-related 7
BBB	blood–brain barrier
BMSCs	bone mesenchymal stem cells
CBF	cerebral blood flow
CBLL1	Cbl proto-oncogene like 1
CCR5	C-C chemokine receptor type 5
CDS	coding sequence
CNS	central nervous system
CPEB2	cytoplasmic polyadenylation element binding protein 2
DRG	dorsal root ganglia
ECM	extracellular matrix
EGFR	epidermal growth factor receptor
EHT	endothelial-to-hematopoietic transition
eIF3	eukaryotic initiation factor 3
FMRP	fragile X messenger ribonucleoprotein
FTO	fat mass and obesity-associated
GDF10	growth differentiation factor 10
GSCs	glioma stem-like cells
HDGF	hepatoma-derived growth factor
HEY2	hairy and enhancer of split-related with YRPW motif 2
HNRNPA2B1	heterogeneous nuclear ribonucleoprotein A2B1
HNRNPC	heterogeneous nuclear ribonucleoproteins C
HNRNPG	heterogeneous nuclear ribonucleoproteins G
HSCs	hematopoietic stem cells
IGF2BP	insulin-like growth factor 2 mRNA-binding protein
LPS	lipopolysaccharide
m6A	N6-methyladenosine
mA	N-Methyl adenosine
MMPs	matrix metalloproteinases
MSCs	mesenchymal stem cells
MVD	micro-vessel density
NPCs	neural progenitor cells
NVU	neurovascular unit
PD	Parkinson’s disease
RBM	RNA binding motifs protein
RBPs	RNA binding-proteins
SAM	S-adenosylmethionine
SCI	spinal cord injury
TBI	traumatic brain injury
TGF β	transforming growth factor β
TPNS	total Panax notoginseng saponin
TRAF6	TNF receptor associated factor 6
TRMT112	tRNA methyltransferase 11-2
VEGF	vascular endothelial growth factor
VIRMA	vir-like m6A methyltransferase-associated protein
VSMC	vascular smooth muscle cell
WTAP	Wilms’ tumor 1-associated protein
YTH	YT521-B homology
